# Physicochemical and Functional Properties of Soluble and Insoluble Dietary Fibers in Whole Grains and Their Health Benefits

**DOI:** 10.3390/foods14142447

**Published:** 2025-07-11

**Authors:** Pathumi Ariyarathna, Patryk Mizera, Jarosław Walkowiak, Krzysztof Dziedzic

**Affiliations:** 1 Department of Agriculture, Sri Lanka School of Agriculture, Dambulla 21100, Sri Lanka; 2 Faculty of Food Science and Nutrition, Poznan University of Life Sciences, Wojska Polskiego 31, 60-624 Poznan, Poland; patrykmizera9@gmail.com; 3 Department of Pediatric Gastroenterology and Metabolic Diseases, Poznan University of Medical Sciences, Szpitalna 27/33, 60-572 Poznan, Poland; jarwalk@ump.edu.pl

**Keywords:** whole grains, dietary fiber, physicochemical properties, non-communicable diseases

## Abstract

The growing global prevalence of non-communicable diseases (NCDs) is drawing an increasing amount of attention to the health-promoting potential of whole-grain dietary fibers. Whole grains are rich sources of both soluble dietary fiber (SDF) and insoluble dietary fiber (IDF), contributing distinct physicochemical properties and playing vital roles in promoting human health. This review provides a comprehensive analysis of the dietary fiber compositions of various whole grains, including wheat, oats, barley, rye, corn, sorghum, and rice, highlighting their structural characteristics, physiochemical properties, and associated health benefits. The physicochemical properties of dietary fibers, such as solubility, water- and oil-holding capacity, viscosity, swelling ability, and bile-acid-binding capacity, contribute significantly to their technological applications and potential health benefits, particularly in the prevention of NCDs. Although there is growing evidence supporting their health benefits, global whole-grain intake remains below recommended levels. Therefore, promoting whole-grain intake and developing fiber-rich functional foods are essential for enhancing public health and preventing chronic diseases. Future research should focus on enhancing the bioavailability and functionality of whole-grain dietary fibers, optimizing the methods by which they are extracted, and exploring their potential applications in the food and pharmaceutical industries.

## 1. Introduction

The rising prevalence of non-communicable diseases (NCDs) is drawing an increasing amount of global attention to healthy dietary choices. Previous studies have shown that an inadequate dietary fiber intake is closely associated with a higher risk of NCDs, including cardiovascular diseases, cancers, gastrointestinal disorders, and type 2 diabetes. Dietary fiber is a member of an edible, non-starch group of polysaccharides that cannot be digested by endogenous enzymes or absorbed in the human small intestine. However, these fibers are partially or completely fermented in the large intestine by beneficial microbiota, thus providing various health benefits [1].

Whole-grain products are considered grain products that contain relative proportions of bran, germ, and endosperm similar to those of untreated grains, and they play a vital role in healthy diets due to their rich content of plant-based dietary fibers. In contrast, refined grains undergo processing that removes the bran and germ, leaving only the starchy endosperm and some proteins, significantly reducing their nutritional composition compared to the original whole-grain products. Whole-grain consumption is associated with numerous health benefits due to these grains’ higher content of dietary fiber, including soluble (β-glucans, arabinoxylans, and pectin) and insoluble fibers (cellulose, hemicellulose, and lignin). These health benefits are largely associated with the physicochemical properties of dietary fibers and their fermentation in the large intestine [2,3].

Epidemiological studies suggest that a daily intake of 50 g of whole grains can lead to significant reductions in health risks, including a 25% lower risk of type 2 diabetes, a 20% reduction in cardiovascular mortality, a 12% decrease in cancer-related mortality, and a 15% reduction in overall mortality [4]. However, guidelines for the recommended intake of whole-grain products vary across the world. According to the US food guide, individuals consuming a 2000-calorie diet are advised to consume approximately 85 g of whole grains per day. Within the European Union, Sweden recommends daily intakes of 90 g for males and 70 g for females, Denmark recommends at least 75 g/day, and Norway suggests 80–90 g/day [2,4]. Despite these recommendations, global whole-grain consumption remains below the recommended levels in many countries. According to the global analysis conducted by Micha et al., the average consumption of whole grains worldwide was approximately 38.4 g/day in 2010, and, notably, only 23 countries reported a mean whole-grain intake greater than 50 g per day, which is considered the minimum threshold for substantial health benefits [5]. According to the Global Dietary Database 2021, the average daily whole-grain intake in Sweden is 78.5 g for males and 80 g for females, while in Denmark, it is approximately 72.1 g for males and 74.2 g for females [6]. The differences between the recommended and actual intakes highlight the need to increase public awareness to improve whole-grain consumption and promote better health outcomes.

While previous studies have extensively focused on the health benefits of whole grains, there is a lack of comprehensive analyses of the different dietary fiber fractions in various whole grains and their physicochemical properties associated with health benefits. Therefore, in this review, we aim to provide a thorough analysis of the dietary fiber compositions of different whole grains, their physicochemical properties, and their functional roles in the prevention of various NCDs. In our study, a comprehensive literature search was conducted by using the PubMed and Google Scholar databases to identify relevant English-language publications published up to April 2025. In our search strategy, we employed a combination of the following keywords: “whole grain dietary fiber,” “soluble dietary fiber,” “insoluble dietary fiber,” “physicochemical properties,” “dietary fiber composition,” “structure of whole grain,” and “health benefits.” Studies were selected based on their relevance to the topic, with a focus on experimental and clinical research published within the last five years, although some older, unique research findings were also included when particularly relevant. Approximately one thousand titles were screened for suitability, and, ultimately, 142 references were selected as the basis for this review.

## 2. Dietary Fiber in Whole Grains

### 2.1. Structure of Whole Grains

**Figure 1:**
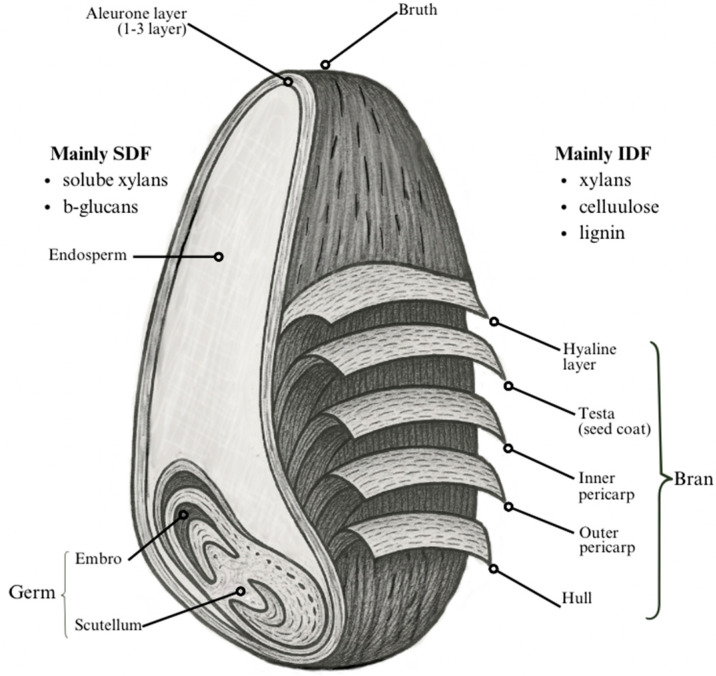
Structure of wheat grain.

According to the Cereals and Grains Association, whole grains consist of the intact, ground, cracked, flaked, or otherwise processed kernel, excluding inedible parts such as the hull and husk. In whole grains, all the anatomical components, including the endosperm, germ, and bran, must be present in the same relative proportions as found in the intact kernel [7]. Examples of whole grains include wheat, rice, maize, oats, rye, barley, triticale, sorghum, and millet, all of which belong to the grass family, Poaceae. Whole grains have a complex structure characterized by multiple cell layers. This structure contains a bark-like protective hull, the endosperm, bran, and germ (Figure 1). The germ contains the plant embryo, while the endosperm provides nourishment for the growing seedling. Surrounding both the germ and the endosperm is the bran, which acts as a protective outer layer of the grain, rich in dietary fiber and bioactive compounds [8].

The endosperm accounts for 80–85% of the whole grain and is primarily composed of starch and protein. The bran and germ represent approximately 12–18% and 2–3% of the dry grain weight, respectively. The embryo is vital for the germination process, as it contains the embryonic axis and scutellum. The endosperm serves as an energy reserve, storing starch and proteins necessary for germination. The aleurone layer, which forms the outermost part of the endosperm, typically consists of one to three layers of cells; this proportion varies among different whole grains. A significant portion of this layer is often removed during roller milling and, therefore, absent in refined flour. The bran fraction consists of several distinct layers, including the outer pericarp, inner pericarp, testa, and nucellar epidermis (hyaline layer). The inner and outer pericarp are rich in polysaccharides, such as cellulose, lignin, and heteroxylan. The testa is a single cell layer in barley, oats, and rice, while in wheat and rye, it generally has two distinct layers [2].

### 2.2. Dietary Fiber Composition of Selected Whole Grains

**Figure 2:**
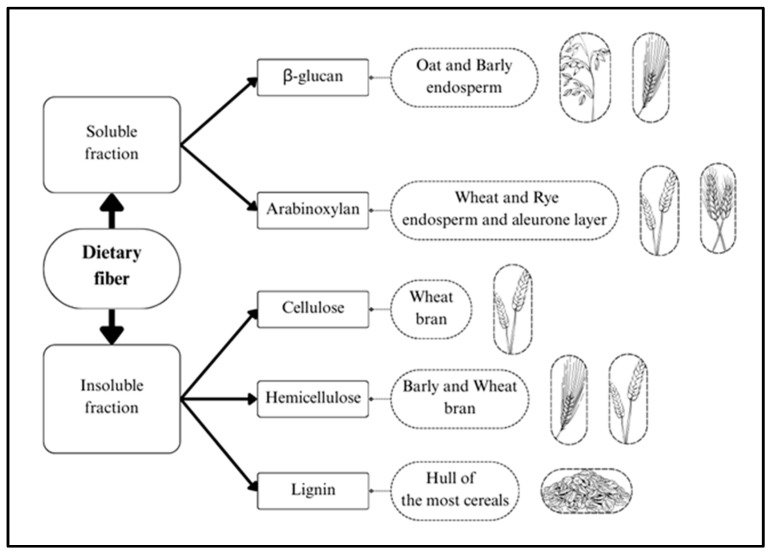
Insoluble and soluble dietary fiber present in whole grains.

Dietary fibers are defined as the edible parts of plants or analogous carbohydrates that resist digestion and absorption in the human small intestine but undergo complete or partial fermentation in the large intestine. Dietary fiber includes polysaccharides (cellulose, hemicellulose, and pectin), oligosaccharides (inulin and other fructo-oligosaccharides), lignin, and other associated plant compounds. It can be classified into two categories based on its water solubility: insoluble dietary fiber (IDF) and soluble dietary fiber (SDF). IDF, which includes cellulose, most hemicelluloses, and lignin, primarily serves as a structural component of plant cell walls. In contrast, SDF comprises various non-cellulosic polysaccharides and oligosaccharides, mainly arabinoxylans and beta-glucans (Figure 2) [7]. Importantly, the ratio of SDF to IDF varies significantly among whole grains and has a substantial influence on the physicochemical properties of dietary fiber, ultimately affecting the grains’ health benefits. For example, oats and barley have relatively higher SDF:IDF ratios (approximately 2:1 to 3:1) due to their richness in β-glucans and arabinoxylan [9,10,11]. In contrast, wheat and rice are predominantly rich in IDF, resulting in lower SDF:IDF ratios (approximately 1:4 to 1:9) [11,12,13,14] (Table S1).

The total dietary fiber (TDF) content of wheat ranges from 9% to approximately 38% on a dry weight basis and includes both insoluble and soluble fractions [2]. In wheat, the fiber in the bran is primarily insoluble, whereas soluble fiber is more prevalent in white flour. The endosperm tissues, including the aleurone and starchy endosperm, contain high proportions of arabinoxylans (60–70%), moderate amounts of (1.3:1.4) β-D-glucans (20–30%), small amounts of cellulose and glucomannan, and no lignin. In contrast, the outer pericarp tissue of wheat has a cell wall composition rich in cellulose (30%), lignin (12%), and xylans (60%), but it contains only limited amounts of β-glucans [2] (Table S1).

In hulless oats, the TDF content typically ranges from 60% to 80%, with approximately 30% comprising SDF, which is primarily located in tissues outside the aleurone layer [15,16]. Oat bran, which accounts for approximately 30–50% of the whole grain, contributes significantly to the dietary fiber content. β-glucan with (1–3), (1–4)-β-D-glucoside linkages is the primary SDF, followed by arabinoxylans. In contrast, lignin and cellulose constitute the predominant IDF fraction [15,16] (Table S1). A previous study investigating 43 oat genotypes reported that dietary fiber content varies significantly by genotype, with some varieties differing by up to 20% in measured fiber content [17,18]. Notably, there are cultivated oat varieties known as hulless oats, which contain quantities similar to those found in other types, but the husk separates much more easily during harvest [15]. Therefore, selecting dietary-fiber-rich oat varieties can offer significant benefits to both food producers and consumers, especially in terms of these varieties’ physicochemical and antioxidant properties [18].

Barley grains with hulls are reported to consist of approximately 20–30% TDF, with β-glucans and arabinoxylans being the most important components [19] (Table S1). SDF, primarily β-glucans, is found in abundance in the endosperm cells, while both β-glucans and arabinoxylans are also present in the aleurone cell walls. Although these compounds are distributed throughout the whole grain, previous studies have shown that arabinoxylans are predominantly concentrated in the outer bran layer and aleurone cells in hulless barley [2,19].

Rye is known for its relatively high dietary fiber content, ranging from 15 to 22% on a dry matter basis [20,21]. The IDF fraction accounts for approximately 10%, while low-molecular-weight SDF, expressed as an indigestible oligomer with a degree of polymerization < 10%, comprises about 5%. High-molecular-weight SDF makes up around 4% [22]. The main non-starch polysaccharides in rye include arabinoxylans, mixed-linkage glucans, arabinogalactans, cellulose, and lignin [20]. Arabinoxylan, a major component of the rye cell wall (constituting approximately 1.3–1.4%), particularly with respect to its water-extractable fraction, plays a significant role in bread dough formation [11,12]. β-glucan is present in rye in lower quantities (1.3–2.2%) compared to those in oats (2.2–7.8%) and barley (3.5–11.3%) [12] (Table S1). The proportions of cellotriosyl and cellotetraosyl units in rye and barley are higher than those in oats, which may influence the functional properties of the SDF fraction [12].

Sorghum has been reported to consist of approximately 19% TDF, which aids digestion by adding bulk to the diet and helping to prevent constipation [23,24]. The dietary fibers in sorghum are primarily located in the cell walls of the pericarp and endosperm. Arabinoxylans and β-glucans are the main types of dietary fiber present, accounting for approximately 55% and 40% of the TDF, respectively. Additionally, the cell wall components of sorghum have been reported to constitute around 20% lignin [25,26] (Table S1).

Corn fiber has been reported to be approximately 70% carbohydrates, including cellulose, xylan, residual starch, and lignin [27]. The composition of corn fiber is typically 25–35% hemicellulose, 16–20% cellulose, 25–35% starch, 2–3% lignin, and 8–10% protein. However, these proportions can vary depending on the corn variety, maturity stage, and processing methods. A key component of the hemicellulose in corn fiber is arabinoxylan, which accounts for about 30% of the hemicellulose content. Corn arabinoxylan is predominantly located in the outer layers of the kernel, such as the bran and pericarp. Arabinoxylans are primarily composed of D-xylose (48–54%) and L-arabinose (33–35%), containing smaller amounts of glucose (7–11%) and glucuronic acid (3–6%). The arabinose-to-xylose ratio, typically around 1:4 or 1:5, plays a crucial role in determining the solubility, functionality, and interactions of arabinoxylan in various applications, including gel formation, and its fermentability by gut microbiota (Table S1). Cellulose, a major structural component of the corn cell wall, and its chemically modified and functionally enhanced forms (microcrystalline cellulose and carboxymethyl cellulose) are widely utilized in the food industry to improve the texture, stability, and mouthfeel of food products [19].

Rice is unofficially categorized into white, brown, and black varieties based on the content and composition of bioactive substances. Brown and black rice varieties contain higher levels of dietary fiber, lipids, and bioactive phytochemicals, including phytosterol-enriched lipids, dark pigments such as anthocyanins, and phenolic compounds with strong antioxidant activity. [12,27]. In rice, dietary fiber is primarily located in the bran. Its content ranges from approximately 0.7–2.7% in white rice to 2.9–4.4% in brown rice and around 6% in unpolished black rice [28,29]. During the milling process, the bran is largely removed, reducing the health benefits of rice while generating food byproducts [30]. It is worth noting that bran, as a main byproduct of rice milling, is composed of 20.5–33.9% dietary fiber, and therefore the most-extracted fraction, which consists of cellulose, hemicellulose, and lignin with small amounts of SDF, is water-insoluble [31,32]. Consequently, numerous studies have analyzed the composition and physical properties of dietary fiber in rice bran [33,34]. White and glutinous rice varieties are reported to have higher proportions of the SDF fraction. The highest SDF content has been found in white rice (16.39%), whereas the highest IDF content has been observed in brown rice (16.51%) [35] (Table S1).

## 3. Physicochemical Properties of Dietary Fiber

Dietary fibers from whole grains exhibit complex structures that significantly influence their various functions in the gastrointestinal tract. These functions are largely determined by their physiochemical properties. The processing methods applied to whole grains can alter both the composition and physicochemical characteristics of dietary fibers. These properties are influenced by various factors, including extraction methods, food sources, molecular structure, and particle size [36,37]. Several researchers have reported a range of physicochemical properties of dietary fiber, including water solubility, water-holding capacity, swelling ability, viscosity, gel-forming ability, bulking capacity, and binding ability [36,37].

### 3.1. Solubility

The solubility of dietary fibers plays a critical role in determining their technological functionality in food systems. Solubility is influenced by several factors, including molecular branching, the presence of ionic groups, and specific positional bonding within the fiber structure. In contrast to insoluble fibers, soluble fibers are known for their ability to increase viscosity and enhance emulsifying properties, making them particularly valuable in food-processing applications [37]. Several studies have highlighted variations in the solubility of dietary fibers from different whole grains. In one study, the solubilities of dietary fiber from oats and wheat were reported to be approximately 3.4% and 4.2%, respectively [38]. Solubility has also been observed to increase during processes such as germination. In whole wheat, for example, solubility has been reported to increase by 18.45 ± 0.23% after germination [38]. The grinding process can alter the solubility of dietary fiber and is often employed as an effective method of enhancing the functional properties of food products. In general, increasing the degree of fiber fragmentation tends to increase the content of SDF while reducing the proportion of IDF [39].

### 3.2. Water-Holding Capacity (WHC)

WHC refers to the amount of water retained by a known weight of dry fiber under defined conditions. The WHC of dietary fibers depends on several factors, including the corresponding molecular structure and the nature of water-binding sites within the fiber matrix [10]. The WHC of dietary fiber is crucial in various food applications, as it significantly influences the shelf life and texture of food products. According to Ahmad et al., β-glucan extracted using hot-water treatment exhibited the highest WHC value (3.79 g/g), followed by that of β-glucan extracted through alkali and acid treatments [40]. In another study, quinoa bran exhibited a higher WHC than oat and rice hulls [41]. Processing methods such as grinding can also affect WHC. For example, the WHC of oats increases from 3.53 to 5.23 g/g with superfine grinding. This improvement is attributed to the increased specific surface area of the sample, thereby enhancing its contact with water [42]. Moreover, in one case, the germination process in whole wheat increased the WHC from 2.12 ± 0.19% to 2.75 ± 0.23%, which is probably related to the increase in the SDF fraction in TDF [38].

### 3.3. Oil-Holding Capacity (OHC)

OHC refers to the amount of oil retained by fibers after mixing and centrifugation. This property is important for minimizing fat loss during cooking and may also aid in the removal of excess fat from the body [10]. Numerous studies have demonstrated the OHC of whole-grain fibers such as quinoa, wheat, oats, and rice bran [43,44]. A previous study found that SDF from quinoa bran SDF exhibited a higher OHC (1.85 ± 0.16 g/g to 2.95 ± 0.23 g/g) than orange peel fiber and oat hulls [41]. Additionally, Dhillon et al. have found that the OHC of whole wheat flour increased following germination, reporting that the OHC of whole wheat flour rose from 2.32 ± 0.21 g/g to 3.78 ± 0.43 g/g after germination [35]. Research conducted by other authors further demonstrates that dietary fibers derived from oats, whole-wheat flour, millet bran, and rice bran possess a high WHC and a low OHC, suggesting their potential as a dietary resource for the development of functional food products that address water synergism in formulated foods and act as emulsifiers for high-fat foods [38,43,44].

### 3.4. Viscosity and Gel Formation

Viscosity (η) refers to a fluid’s resistance to flow and is mathematically defined as the ratio of shear stress (τ) to shear rate ($\dot{\gamma }$). Viscosity plays a significant role in increasing enteric viscosity, which can slow intestinal transit, delay gastric emptying, and reduce the absorption of glucose and sterols in the intestine. These effects help lower serum cholesterol, postprandial blood glucose, and insulin levels. These health benefits are primarily associated with viscous SDF [45,46]. In whole grains, SDF plays a crucial role in enhancing the viscosity of solutions. In contrast, insoluble fibers, such as cellulose found in rice bran and wheat bran, generally contribute less to viscosity due to their inability to dissolve in water and form gel-like structures [46].

A previous study found that oat dietary fiber, particularly β-glucan, has the ability to reduce glycemic responses, primarily due to its role in increasing viscosity within the upper digestive tract. The viscosity of β-glucan samples ranged from 34.30 to 52.82 cP, depending on the extraction method used. The highest viscosity (52.82 cP) was observed in enzymatically extracted β-glucan, while the lowest viscosity was found in alkali-extracted β-glucan. Additionally, factors such as the temperature and pH of the solution were also found to significantly influence the viscosity of β-glucan solutions [40]. Soluble fibers such as β-glucan form gels that increase the viscosity of gastrointestinal contents. For example, long-chain polymers like guar gum and tragacanth gum retain large amounts of water and exhibit high viscosity, whereas highly soluble fibers like gum arabic display low viscosity despite their solubility [46].

### 3.5. Bile-Acid-Binding Capacity

Cholesterol serves as a precursor for bile acid synthesis in the liver. Dietary fibers can bind to bile acids, promoting their excretion from the body. This loss stimulates the conversion of more cholesterol into bile acids, thereby lowering blood cholesterol levels and reducing the risk of heart disease [29]. The gel formed by SDF in the presence of Ca^2+^ can encapsulate bile acids. Various water-soluble polysaccharides contribute to bile salt binding, primarily due to the presence of functional groups such as –COH, –C=O, –O–, –COOH, and –OH, which exhibit high affinity for cholesterol and bile salt structures. Additionally, lignin—a highly branched component of IDF—also plays a significant role in this mechanism, particularly due to its abundance of hydroxyl and carbonyl groups [47].

A previous study reported that the bile-acid-binding capacities of dietary fiber from the rice cultivars Mushq Budij and SR−4 were 18.10 μmol/g and 23.24 μmol/g, respectively [29]. A study by Liu et al. explored the bile-acid-binding capacity of soluble dietary fiber extracted from various colored quinoa varieties [41]. Bile acids such as cholic acid (CA) and chenodeoxycholic acid (CDCA) play a vital role in lipid metabolism. The cited study found no significant differences in CA adsorption capacity among the different SDF samples extracted from quinoa. However, the adsorption capacity for CDCA varied significantly among the samples [41].

### 3.6. Swelling Ability

The swelling ability of dietary fiber refers to their capacity to expand or gelatinize when mixed with water and is expressed as a ratio of the fibers’ volume after being soaked in water to their original weight. This ability varies depending on the solubility, chemical structure, and source of the dietary fiber as well as additional factors such as temperature and ionic charge [48]. According to a previous study, the swelling ability of wheat bran dietary fiber increased from 2.14 to 2.60 mL/g after it was ground. This improvement may be attributed to structural changes in the dietary fiber induced by the grinding process [49]. In another study, the swelling ability of oat bran dietary fiber increased significantly (*p* < 0.05) with extended grinding times. This enhancement is likely due to the superfine grinding process, which increases the specific surface area of a sample, thereby increasing its contact with water [42]. Other authors have reported that extraction methods involving the use of alkaline and acidic treatments significantly affect the water-swelling ability of dietary fiber. In both cases, the fibers extracted using alkaline treatment—both SDF and IDF—exhibited the highest swelling values. This result was attributed, in the case of SDF, to a loose microstructure, a reduced particle size, and, consequently, a larger surface area for interaction with water molecules. For IDF, strong alkali treatment breaks down cellulose chains into shorter polymers and disrupts the ether linkages between hemicellulose and lignin, enhancing water absorption capacity [48]. In food processing, enhanced swelling ability improves functional performance and can better meet the growing public demand for health-oriented food products.

## 4. Health Benefits of Whole-Grain Dietary Fiber

**Figure 3:**
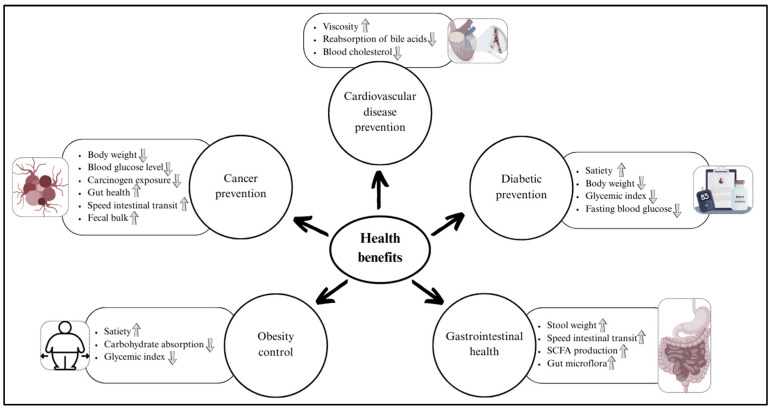
Health benefits of whole-grain dietary fibers.

The physicochemical properties of dietary fibers, including their solubility, water- and oil-holding capacity, swelling ability, viscosity, gel-forming capacity, and bile-acid-binding potential, play important roles in determining their biological functionality and related health benefits. This section focuses on explaining how specific types of dietary fibers contribute to the prevention and management of NCDs such as cardiovascular disease, type 2 diabetes mellitus, obesity, gastrointestinal disorders, and certain cancers. Figure 3 presents the health benefits of whole grains in relation to the physicochemical characteristics of their fiber components. Moreover, a detailed summary of previous studies investigating the health effects of whole-grain dietary fibers is provided in Table 1 at the end of this section.

### 4.1. Prevention of Cardiovascular Disease

Elevated low-density lipoprotein cholesterol (LDL-C) levels and postprandial hyperglycemia are key risk factors contributing to the development of atherosclerotic cardiovascular disease (CVD). While synthetic medications such as statins are effective in managing CVD, their long-term use can be associated with high costs and potential side effects, particularly when consumed in large doses over extended periods [50]. Consequently, dietary fibers are receiving growing attention for their potential health benefits, especially in the prevention and management of CVD (Figure 3). A substantial body of research has examined the protective effects dietary fibers derived from whole-grain cereals have against CVD, highlighting their role in modulating lipid profiles, reducing blood glucose levels, and improving overall cardiovascular health [51,52,53] (Table 1). Compared to IDF, SDF is more frequently associated with reductions in serum low-density lipoprotein cholesterol (LDL-C) levels, and it is often linked to a lower risk of CVD. Elevated viscosity caused by SDF impedes the reabsorption of bile acids, thereby promoting their excretion and reducing the enterohepatic circulation of cholesterol. As a result, hepatic cholesterol is redirected for bile acid synthesis, contributing to a decline in circulating cholesterol levels [54].

A cohort study involving 7469 participants with CVD demonstrated that increased dietary fiber intake was associated with significant reductions in both total cholesterol and LDL levels. Specifically, total cholesterol levels decreased by 0.42 mmol/L (95% CI: −0.78 to −0.05), while LDL cholesterol levels decreased by 0.47 mmol/L (95% CI: −0.85 to −0.10) [55]. Moreover, several studies have demonstrated the potential of oats and oat-based products to improve blood lipid profiles and lower blood pressure by modulating insulin metabolism in individuals with mild hypercholesterolemia [56,57,58]. β-glucans present in oats have been shown to reduce cholesterol absorption in the small intestine by forming a viscous gel that binds to cholesterol molecules [59]. Large-scale meta-analyses of randomized controlled trials and epidemiological studies have concluded that daily consumption of 3 g of oat or barley β-glucans results in significant reductions in total and LDL cholesterol levels in both healthy and hypercholesterolemic individuals [60]. Additionally, a previous study reported that oat bran dietary fiber supplementation was associated with a reduction in heart rate among patients with hypertension [57]. Experimental studies on mice have demonstrated that enzyme-treated wheat bran—rich in arabinoxylans, cellulose, and lignin—significantly reduced body weight and hepatic triglyceride content when administered to five-week-old mice [61].

Soluble fiber is suggested to reduce cholesterol synthesis by modulating serum concentrations of hormones and short-chain fatty acids (SCFAs), such as acetate, propionate, and butyrate, which influence lipid metabolism. Notably, butyrate was shown to significantly inhibit de novo cholesterol synthesis in isolated rat hepatocytes [62]. These findings suggest that increasing dietary fiber intake, particularly from whole-grain sources, may represent an effective nutritional strategy for the prevention and management of CVD.

Moreover, hypertension, defined as a systolic/diastolic blood pressure level exceeding 140/90 mmHg, is a significant risk factor for cardiovascular disease (CVD) [63]. Recent research has highlighted the protective role of whole-grain dietary fibers in blood pressure regulation and vascular health. A meta-analysis involving 7469 adults with CVD demonstrated that increasing dietary fiber intake significantly reduced systolic blood pressure by 4.3 mmHg (95% CI: 2.2 to 5.8) and diastolic pressure by 3.1 mmHg (95% CI: 1.7 to 4.4) [55]. Another study recommended a minimum daily fiber intake of >28 g/day for hypertensive women and >38 g/day for hypertensive men, estimating that each additional 5 g/day reduces systolic and diastolic blood pressure (BP) by 2.8 mmHg and 2.1 mmHg, respectively [64].

Mechanistically, soluble fibers may reduce BP by lowering insulin resistance and serum insulin levels. Another emerging mechanism involves the modulation of gut microbiota composition through fiber intake. Microbiota-derived metabolites such as short-chain fatty acids (SCFAs), indole-3-lactic acid (beneficial), and trimethylamine N-oxide (TMAO, unfavorable) have been shown to interact with gene pathways involved in BP, regulation, including those affecting the renin–angiotensin–aldosterone system (RAAS) [65]. Furthermore, the gut microbiota can influence sympathetic nervous system tone by triggering intestinal chromaffin cells to secrete neurotransmitters such as serotonin, dopamine, and norepinephrine, which may also impact vascular tone and BP levels [66].

In a recent in vivo study, oral administration of β-glucans (100 mg/day) was found to upregulate the expression of corin. Corin is a cardiac protease (an ANP-converting enzyme) that activates natriuretic peptides [63]. This corin–ANP pathway plays a crucial role in sodium–water balance and BP reduction. These cardioprotective effects are largely mediated through the fermentation of soluble fibers such as β-glucans (abundant in oats and barley), which are converted by gut microbiota into SCFAs, including acetate, propionate, and butyrate. SCFAs are readily absorbed by intestinal epithelial cells and engage in pleiotropic actions: they inhibit HMG-CoA reductase (the rate-limiting enzyme of cholesterol biosynthesis), reduce systemic inflammation, and protect vascular function [63]. Importantly, SCFAs differentially regulate BP and vascular tone through the activation of two G-protein coupled receptors—GPR41 and Olfactory Receptor 78 (Olfr78). The activation of Olfr78 by acetate and propionate promotes renin release and vasoconstriction, increasing BP. In contrast, SCFA binding to GPR41 lowers BP by reducing vascular resistance. This dual signaling creates a protective buffering system that stabilizes BP during physiological fluctuations [67,68].

### 4.2. Prevention of Type 2 Diabetes Mellitus

Diabetes mellitus is a non-communicable metabolic disorder characterized by the inability to produce or effectively utilize insulin. Type 2 diabetes mellitus (T2DM), which results from inadequate insulin production or insulin resistance, accounts for approximately 90% of diabetes cases in the adult population [2]. Whole-grain dietary fibers play a critical role in the prevention of T2DM, largely due to their association with a low glycemic index, increased satiety, and reduced body weight (Figure 3). Regular consumption of at least 15.9 g of dietary fiber per day has been shown to improve glycemic control by lowering fasting blood glucose levels, postprandial insulin concentrations, and serum triglyceride levels [69].

This elevated viscosity significantly contributes to reduced glucose absorption by slowing gastric emptying and decreasing the rate of starch digestion, thereby delaying the postprandial rise in blood glucose levels [2,70] (Table 1). The increased viscosity induced by SDF may also result in the formation of a ‘thick layer’ surrounding food particles, thereby limiting digestive enzymes’ ability to access the inner components of the food matrix and delaying their interaction with the absorptive surfaces of the gastrointestinal tract [2]. SDF plays a vital role in the management of T2DM, whereas IDF does not directly impact postprandial glucose levels or insulin sensitivity in T2DM [70]. However, some studies suggest that IDF may also offer significant potential in reducing the risk of T2DM [70,71]. The beneficial effects of IDF may be attributed to its fermentation by gut microbiota and the subsequent production of bioactive compounds. IDF also acts as a physical barrier, slowing the transit of digestive products across the enterocyte brush border and modulating the activity of digestive enzymes. Additionally, microbial fermentation releases phenolic compounds bound to the fiber matrix, thereby increasing the bioavailability of these phytochemicals. Polyphenols, recognized as potent bioactive compounds, are considered a promising alternative strategy for the prevention and management of T2DM [70].

A higher glycemic index (GI) has been linked to an increased risk of T2DM. Previous studies have reported average GI values for various whole-grain products, including 42 for whole-grain cold breakfast cereals, 55 for oatmeal and brown rice, and a range of 27 to 70 for whole-grain bread. This variability in GI values for whole-grain breads depends largely on the main ingredients used, such as barley, buckwheat, oats, and rye [72]. Furthermore, a comprehensive systematic review of eleven prospective cohort studies, encompassing 463,282 participants and 37,249 type 2 diabetes cases, found that a higher daily intake of whole grains was associated with a reduced risk of developing T2DM [73]. Specifically, a higher oat intake (more than 5.7 g/day) was significantly associated with a lower risk of T2DM [74]. Oat-based products, such as oat flakes, oatmeal, and oat bran, generally have a lower glycemic index than similar products made from wheat, barley, or corn. This effect is largely attributed to β-glucans, the primary components of oat fiber. β-glucan is a water-soluble, non-digestible polysaccharide that is not absorbed in the small intestine. By increasing the viscosity of the alimentary bolus in the upper gastrointestinal tract, β-glucan slows nutrient absorption and reduces postprandial glucose spikes [74]. Similarly, dietary fiber from millet bran has demonstrated the ability to reduce fasting blood glucose (FBG) levels in rat models, further highlighting the potential of whole-grain fibers in glycemic control [75]. Wheat bran dietary fibers have also demonstrated significant benefits in diabetic models by increasing serum insulin content. Specifically, intake of modified wheat bran dietary fibers significantly elevated serum insulin levels and appeared to inhibit hepatic gluconeogenesis by suppressing the 1,2-DAG-PKCε signaling pathway. This suppression enhanced insulin receptor activity and insulin signal transduction, collectively resulting in reduced blood glucose levels in diabetic mice [76].

### 4.3. Control of Obesity

Obesity is a major global health concern, impacting both developed and developing countries and significantly increasing the risk of CVD, T2DM, and certain types of cancer [77]. According to the World Health Organization (WHO), more than 1 billion people worldwide were living with obesity in 2023, including approximately 650 million adults, 340 million adolescents, and 39 million children [78]. The primary cause of obesity is an imbalance between energy intake and expenditure, driven by excessive calorie consumption via food and beverages combined with insufficient physical activity. Effective obesity management requires creating a negative energy balance, which reduces fat stores while preserving lean body mass. Treatment plans should be personalized, taking into account factors such as age, sex, degree of obesity, and individual health risks. There are various approaches to tackling this global epidemic, with lifestyle modification playing a central role in all weight management strategies. Behavioral changes that promote self-control of daily energy balance through diet and physical activities are essential for long-term success and, importantly, maintaining body weight [79,80].

Compared to refined grains, whole grains contain fewer starches and calories but are richer in micronutrients and phytochemicals, offering significant health benefits [81]. Previous studies have shown significant inverse associations between whole-grain fiber intake and measures of body weight [82,83] (Table 1). A previous meta-analysis found that higher whole-grain intake was significantly associated with lower body mass index (BMI) values, showing a weighted slope of −0.0141 kg/m^2^ per gram per day (95% CI: −0.0207 to −0.0077; r = −0.526, *p* = 0.0001) [84]. Similarly, another study reported a significant reduction in BMI and fat mass among overweight and obese adults after a whole-grain diet intervention over a defined period [85]. A 2019 meta-analysis of observational studies and randomized controlled trials found that higher whole-grain consumption is significantly associated with lower BMI values. Analysis of cross-sectional data from 12 studies, involving 136,834 participants, indicated a significant inverse correlation between whole-grain intake and BMI [84]. In another study involving 5094 randomly selected Finnish adult men, a significant inverse association was observed between whole-grain intake and BMI (*p* < 0.001), waist circumference (*p* < 0.001), and total cholesterol levels (*p* = 0.02) [82].

The naturally high fiber content in most whole-grain foods may help prevent weight gain by enhancing appetite control and slowing carbohydrate absorption. Additionally, the presence of multiple enzyme inhibitors within the whole-grain fiber matrix may directly influence metabolic efficiency, providing another mechanism through which whole grains can positively impact body weight (Figure 3). Conversely, the high insulin concentrations often associated with consuming low-fiber refined grains may, over the long term, promote weight gain by shifting metabolic fuels from oxidation to storage [86,87].

### 4.4. Gastrointestinal Health

Whole grains are an important source of dietary fiber, which exerts prebiotic effects by selectively stimulating the growth and activity of beneficial gut microbiota, thereby contributing to improved gastrointestinal health and immune function. In addition to their prebiotic activity, dietary fibers positively impact digestive health by increasing stool bulk, accelerating intestinal transit, promoting the production of short-chain fatty acids (SCFAs), and modulating gut microbiota composition [3] (Figure 3). Recent studies have demonstrated that β-glucan consumption enhances the production of SCFAs and positively modulates gut microbiota composition. For example, Thandapilly et al. reported that high-molecular-weight barley β-glucan significantly increased fecal SCFA concentrations in individuals with mild hypercholesterolemia [88]. Additionally, Pi et al. found that hydrolyzed β-glucan promoted the production of SCFAs and gases, also increasing the abundance of beneficial bacteria such as Bifidobacterium and Faecalibacterium, suggesting its potential role in improving gut health and modulating the microbiota [89]. Previous research has shown that whole-grain oats, barley, and their β-glucans generally exhibit prebiotic properties by promoting an overall increase in colonic microbial populations and activity, particularly favoring *Lactobacillus acidophilus* and *Bifidobacterium longum* [90]. These studies also observed an increase in SCFA production using porcine and mouse models [91,92]. In another study involving 31 adults with overweight and class-I obesity, supplementation with corn arabinoxylan was found to modulate gut microbiota composition by promoting the growth of *Bifidobacterium longum*, *Blautia obeum*, and *Prevotella copri*. Additionally, it increased fecal propionate concentrations, which are known for their potential metabolic benefits. Compared to arabinoxylans from other sources such as rice, corn, and wheat, corn arabinoxylan yielded the highest quantities of health-beneficial SCFAs [19].

Bifidobacteria and Lactobacilli are key gut microorganisms associated with significant health benefits. Bifidobacterium has been shown to protect against diseases and conditions such as colorectal cancer, diarrhea, necrotizing enterocolitis, and inflammatory bowel disease (IBD). It also competitively inhibits pathogens from attaching to epithelial cell binding sites. Lactobacillus plays a protective role in maintaining intestinal barrier integrity by mitigating inflammation, chemical damage, and stress-induced permeability. Additionally, Lactobacillus produces lactate, which is further metabolized into SCFAs. Bacterial fermentation of dietary fibers in the colon generates SCFAs, including acetate, propionate, and butyrate. Bifidobacterium contributes to SCFA production by generating acetate and maintaining a symbiotic relationship with butyrate-producing bacteria. Consumption of dietary fibers, such as those from whole grains, enriches butyrate levels, which are vital for maintaining colonocyte health and promoting apoptosis in mutated colon cells, thereby supporting colon health and potentially reducing cancer risk [88,93].

Recent studies have confirmed the beneficial effects of whole grains on bowel health. A randomized controlled trial demonstrated that a diet rich in whole-grain fiber enhances fecal bulk and colonic fermentation, resulting in increased defecation frequency and higher fecal water content—both of which are indicators of improved bowel function [94]. Additionally, research on barley β-glucan consumption has shown that it increases fecal bile acid excretion and elevates production of SCFAs, including butyrate.

Rye-based foods have been reported to be more effective than whole-wheat and low-fiber foods in increasing plasma enterolactone and fecal butyrate concentrations. Elevated enterolactone levels result from the beneficial microbial fermentation of mammalian lignans in the gut lumen, followed by their absorption through the colon wall. Enterolactone is considered a potential biomarker for large-bowel health [95]. Additionally, fecal ammonia and p-cresol, potentially toxic compounds produced in the large bowel, can negatively impact colon wall health. Whole-grain dietary fibers have been shown to reduce the colon’s exposure to these harmful substances, thereby promoting improved large-bowel health [96].

Dietary fiber, which is especially rich in phenolic compounds like ferulic acid, is abundant in the bran of the wheat kernel and is released through microbial fermentation in the colon. Ferulic acid in plasma or feces serves as a biomarker for bacterial fermentation activity in the colon [97,98,99]. Additionally, a controlled trial reported the beneficial effects of consuming 70 g of whole-grain wheat (providing 8 g of fiber) daily for 8 weeks compared to those yielded by 60 g of refined wheat (providing 2.2 g of fiber). The study demonstrated significant increases in ferulic acid levels, with a 4-fold rise in plasma concentrations and a 2-fold increase in fecal excretion in the whole-grain group compared to the group of refined wheat consumers [98].

### 4.5. Prevention of Cancers

Several studies have suggested that whole-grain fibers help protect the body against various types of cancer, including gastric, colorectal, breast, and prostate cancers [100,101,102,103] (Table 1). Moreover, a higher whole-grain intake is inversely associated with weight gain and the risk of T2DM, both of which are established risk factors for cancer development [104]. Moreover, the dietary fibers in whole grains increase fecal bulk and reduce intestinal transit time, thereby helping dilute carcinogens and limit their absorption by the intestinal epithelium (Figure 3). Resistant starches and oligosaccharides present in whole grains are fermented in the colon to produce SCFAs, including butyrate. These SCFAs serve as a preferred energy source for mucosal cells and exhibit proapoptotic and antineoplastic properties that help inhibit tumor growth. Additionally, SCFAs lower the intestinal pH, reducing the solubility of free bile acids and thereby decreasing their availability for carcinogenic activity [94,105]. Therefore, dietary fibers in whole grains play a vital role in protecting the body against various types of cancer.

A previous study found that participants in the highest quintile regarding whole-grain intake had a 22% lower risk of developing liver cancer [106]. Another study demonstrated that barley exhibited anti-tumor activities in both rat mammary tumor models and MCF-7 breast cancer cell lines through mechanisms including the induction of cell cycle arrest, the promotion of apoptosis, and the inhibition of cell proliferation [97,98]. Numerous studies have documented the anticancer potential of oat β-glucans, demonstrating their effectiveness against various cancer cell lines, both in vitro and in vivo [107,108]. Another study suggested that oat β-glucans are a safe and effective option for managing early-stage colorectal cancer [109].

Dietary fibers in whole grains play a significant role in colorectal cancer prevention, largely due to their interactions with bile acids. Bile acids are amphiphilic molecules essential for lipid absorption in the small intestine and the formation of micelles that solubilize cholesterol. In the large intestine, gut bacteria metabolize bile acids by deconjugating and dehydroxylating primary bile acids to form secondary bile acid compounds that have been implicated in colonic carcinogenesis. Several studies have demonstrated that these bile acids are cytotoxic to colonocytes and can stimulate abnormal cell proliferation. Dietary fibers bind bile acids, thereby modifying the gut–liver axis, reducing circulating cholesterol levels, and consequently decreasing the risk of colorectal cancer development [110].

**Table 1:** Health benefits of different whole grains.

Dietary Fiber Source	Study Type	Health Benefit	Research Findings	Reference
Rye	Human intervention	Cardiovascular disease prevention	Total and LDL cholesterol levels were lower (−0.06 and −0.09 mmol/L, respectively; *p* < 0.05) after patients had consumed whole-grain rye with lignan supplements for 4 weeks.	[111]
		Diabetic control	Rye kernel bread decreased blood glucose levels (0–120 min, *p* = 0.001), serum insulin response time (0–120 min, *p* < 0.05), and fasting FFA concentrations (*p* < 0.05).	[112]
			Rye-based foods decreased postprandial glucose and insulin responses.	[113]
		Obesity control	Participants who consumed a rye-based diet for the 12-week period lost 1.08 kg of body weight and 0.54% more body fat than the group that consumed refined wheat (95% confidence interval (CI): 0.36; 1.80, *p* < 0.01 and 0.05; 1.03, *p* = 0.03, respectively).	[114]
		Gastrointestinal health	Induced some changes in gut microbiota composition, including increased abundance of the butyrate-producing *Agathobacter*.	[115]
Oat	Human intervention	Cardiovascular disease prevention	There was a significant reduction in office systolic blood pressure (oSBP; *p* < 0.001) and office diastolic blood pressure (oDBP; *p* < 0.028) in the group that consumed oat bran (30 g/day of oat bran contains 8.9 g of dietary fiber) compared to the control group after a 3-month period.	[58]
			Consumption of oat dietary fiber reduces levels of systemic chronic inflammation after two weeks post-treatment.	[116]
		Diabetic control	Adhering to a diet enriched with 5 g of oat β glucan for 12 weeks can help improve glycemic control, increase the feeling of satiety, and promote changes in the gut microbiota profile.	[117]
			The results demonstrated that a hypocaloric oat-based diet led to a significant reduction in total insulin dosage and HbA1c levels in insulin-treated outpatients with type 2 diabetes.	[118]
		Obesity control	Oat β-glucan intervention increased the abundance of Lactobacillus and Bifidobacterium. These microbiota alterations contributed to an increase in 7-ketodeoxycholic acid levels and enhanced bile acid synthesis.	[119]
		Ageing control	A decrease in levels of the protein Eotaxin-1, an aging-related chemokine, independent of a person’s gender, body mass index, or age.	[116]
Wheat	Human intervention	Cardiovascular disease prevention	Total and LDL cholesterol levels in 40 men with a metabolic syndrome risk profile were lowered by −0.09 mmol/L (at *p* < 0.05) after they had consumed a wheat-based diet for 4 weeks.	[111]
		Obesity control	The study found that consumption of resistant starch-enriched wheat rolls significantly increased fasting and peak concentrations of peptide YY3–36 (PYY3–36), a hormone associated with satiety, while decreasing peak concentrations and iAUC of glucose-dependent insulinotropic peptide (GIP), which is involved in hunger regulation.	[120]
		Gastrointestinal health	The study found that consuming 15 g/day of wheat bran extract increases fecal Bifidobacterium quantities and softens stool without having major effects on energy metabolism in healthy humans with slow GI transit.	[121]
	In vivo study	Gastrointestinal health	The study found that high amylose wheat (HAW) consumption led to an increase in fecal bacterial loads and gastrointestinal health in mice.	[122]
Corn	Human intervention	Cardiovascular disease prevention	Whole-grain corn flour significantly decreased LDL cholesterol levels over time (−10.4 ± 3.6 mg/dL, *p* = 0.005) and marginally decreased total cholesterol levels (−9.2 ± 3.9 mg/dL, *p* = 0.072) over time.	[123]
Brown rice	Human intervention	Diabetic control	The researchers observed improved endothelial function, without changes in HbA1c levels	[124]
Whole grains	Human intervention	Diabetic control	Higher intake of whole-grain fiber was positively associated with better β-cell function, insulin sensitivity, and postprandial glycemic control.	[125]
			A systematic review found that increasing whole-grain fiber intake improves glycemic control and reduces cardiometabolic risk factors in individuals with prediabetes, type 1, or type 2 diabetes. The results obtained suggest increasing daily fiber intake by 15 g or to a total of 35 g per day can lower the risk of premature mortality and enhance diabetes management.	[126]

## 5. Strategies for Enhancing the Physiochemical and Functional Properties of Whole-Grain Dietary Fiber

Recent advances in food-processing and pre-treatment technologies offer promising strategies for enhancing the physiochemical and health-promoting properties of whole-grain dietary fibers (DFs). These approaches are designed to modify fiber structure, improve functionality, modulate the soluble/insoluble fiber (SDF/IDF) ratio, and liberate bioactive compounds (polyphenols) that are often bonded to the fiber matrix.

Germination is one of the most effective bioprocessing methods used to enhance the nutrient compositions and functional properties of whole grains. During germination, endogenous enzymes are activated, modifying the plant cell wall structure and increasing the solubility of dietary fibers. Studies have shown that germination leads to a significant increase in SDF levels while decreasing IDF levels, although the total dietary fiber (TDF) content remains constant or slightly decreases during the initial 4–6 days [127]. For example, 48 h germination improved the fiber content of wheat, millet, and maize by 14.4%, 62%, and 21.4%, respectively [128]. Similarly, another study reported a reduction in TDF and IDF levels in whole-grain germinated corn flour from 19.1 ± 1.4 to 17.2 ± 1.1 g/100 g and from 18.1 ± 1.3 to 16.1 ± 0.4 g/100 g, respectively, while SDF levels increased from 1.2 ± 0.2 to 1.9 ± 0.2 g/100 g [129].

Extrusion is a versatile food-processing technique that combines mixing, cooking, cutting, and forming operations and is commonly used in the production of baby foods, grain breakfast products, and pre-cooked flour. The desired product characteristics can be achieved by adjusting feed moisture, machine temperature, screw speed, pressure, screw diameter, and other extrusion parameters, thereby potentially enhancing the functional and physiochemical properties of dietary fiber in whole grains. Studies have reported that extrusion reduces IDF content and increases SDF content, thereby enhancing fiber fermentability and health potential. This transformation is due to the mechanical shear and heat applied during extrusion, which break down chemical bonds in IDF, converting them into soluble forms [130]. Boakye et al. (2023) found that extrusion at 20% feed moisture, a screw speed of 300 rpm, and an extrusion temperature of 150 °C reduced IDF content by 40% and increased SDF content by 74% [131]. Similar findings were observed for whole-grain corn flour, with extrusion reducing TDF content from 19.1 ± 1.4 g/100 g to 17.9 ± 0.7 g/100 g and IDF content from 18.1 ± 1.3 g/100 g to 15.5 ± 0.6 g/100 g while also increasing SDF from 1.2 ± 0.2 g/100 g to 2.2 ± 0.3 g/100 g [129].

Sourdough fermentation, a traditional bioprocess involving lactic acid bacteria (LAB) and yeast (mainly *Saccharomyces cerevisiae*), has been widely utilized in cereal-based product development [132]. Fermentation of wheat bran IDF using LAB (*L. plantarum*) enhanced water retention capacity (WRC), oil retention capacity (ORC), and water-swelling capacity (WSC). It also improved antioxidant activity, as shown via DPPH, ABTS, and reducing power assays [133]. Similarly, millet bran DF through fermentation with *Bacillus natto* increased the proportion of SDF and improved the adsorption capacity for cholesterol, bile salts, nitrites, and glucose [134]. In oat bran, fermentation with *Saccharomyces cerevisiae* increased TDF content from 24.21 ± 0.9% to 28.49 ± 1.3%, SDF content from 5.01 ± 0.02% to 7.2 ± 0.02%, and IDF content from 19.2 ± 0.1% to 21.31 ± 0.2% [135].

However, enzymatic degradation during sourdough fermentation using oat and barley flour can reduce the content of β-glucan by 10–30%, although its solubility may increase. Strategies such as adding oat or barley ingredients later in the process or using coarse flour with large particles to slow hydration help preserve β-glucan integrity [136]. Moreover, a previous study revealed that producing sourdough with wheat flour combined with oat bran increased the molecular weight of β-glucan, potentially due to the partial inactivation of degrading enzymes during fermentation [136].

## 6. Conclusions

Whole-grain dietary fibers exhibit a wide range of physicochemical properties, including solubility, water- and oil-holding capacity, swelling ability, viscosity, gel formation, and bile-acid-binding capacity, which contribute not only to their technological utility but also their role in preventing non-communicable diseases (NCDs). The dietary fiber content and composition vary significantly among different whole grains, such as wheat, oats, barley, rye, corn, sorghum, and rice. Evidence from in vitro, in vivo, and clinical studies highlights the beneficial effects of whole-grain dietary fibers against various NCDs, including cardiovascular diseases, type 2 diabetes mellitus, obesity, gastrointestinal disorders, and certain cancers, through different mechanisms. Among the studied whole grains, oats and barley have exhibited the most-pronounced health benefits, largely due to their high content of soluble dietary fibers (SDFs), especially β-glucans and arabinoxylans. These SDFs are highly fermentable, capable of forming gels in the gastrointestinal tract, and are reported to be able to lower serum LDL cholesterol levels, improve insulin sensitivity, modulate gut microbiota, and reduce systemic inflammation. In contrast, wheat, sorghum, corn, rice, and rye, which are predominantly rich in insoluble dietary fibers (IDFs) such as cellulose, hemicellulose, and lignin, have shown significant benefits in promoting bowel regularity, enhancing satiety, and supporting gut health through microbial fermentation and the production of short-chain fatty acids (SCFAs). Dietary guidelines for the recommended intake of whole-grain products vary across the world, and epidemiological studies suggest that consuming at least 50 g of whole grains can lead to significant reductions in health risks, including a 25% lower risk of type 2 diabetes, a 20% reduction in cardiovascular mortality, a 12% decrease in cancer-related mortality, and a 15% reduction in overall mortality. In addition, several meta-analyses suggest that a daily intake of at least 3 g of β-glucans from oats or barley can help significantly reduce total and LDL cholesterol levels. Despite strong scientific evidence, the global intake of whole grains remains below the recommended levels. Therefore, promoting whole-grain intake and developing fiber-rich functional foods are essential for enhancing public health and preventing chronic diseases. Future research should focus on enhancing the bioavailability and functionality of whole-grain dietary fibers, optimizing the methods by which they are extracted, and exploring their potential applications in the food and pharmaceutical industries.

## Data Availability

No new data were created or analyzed in this study.

## Supplementary Materials

The following supporting information can be downloaded at https://www.mdpi.com/article/10.3390/foods14142447/s1. Table S1: Dietary fiber compositions of different whole grains.

## Abbreviations

The following abbreviations are used in this manuscript:

NCDs	Non-Communicable Diseases
IDF	Insoluble Dietary Fiber
SDF	Soluble Dietary Fiber
TDF	Total Dietary Fiber
WHC	Water-Holding Capacity
OHC	Oil-Holding Capacity
CA	Cholic Acid
CDCA	Chenodeoxycholic Acid
LDL-C	Low-Density Lipoprotein Cholesterol
CVD	Cardiovascular Disease
SCFAs	Short-Chain Fatty Acids
T2DM	Type 2 Diabetes Mellitus
GI	Glycemic Index
WHO	World Health Organization
BMIBody	Mass Index
IBD	Inflammatory Bowel Disease
