# Supplementary material for: Physicochemical and Functional Properties of Soluble and Insoluble Dietary Fibers in Whole Grains and Their Health Benefits
Source: Foods. 2025 Jul 11;14(14):2447. doi: 10.3390/foods14142447 (PMC12295008; doi:10.3390/foods14142447)
Supplement: Supplementary file 1 [file foods-14-02447-s001.zip › foods-3699890-supplementary.pdf]

**Table S1**. Dietary fiber composition of different whole grains.

| Crop | DF content (%) | Cellulose (%) | Hemicellulose (%) | Lignin (%) | β-glucan (%) | Arabinoxylan (%) | References |
|---|---|---|---|---|---|---|---|
| **Whole Wheat** | 10.2–15.7 | 1.40–3.00 | 6.70–9.00 | 1.80–2.87 | 0.60 | 1.64–4.38 | [1–9] |
| Bran | 85.0–93.0 | 25.0–32.0 | 29.0–33.0 | 10.0–17.4 | 1.00–6.00 | 11.0–16.4 (including water soluble 3.00–5.00) | |
| Endosperm | 4.12–4.47 | 0.30 | 1.00< | – | 0.30–0.42 | 1.52–1.75 (including water soluble 0.56) | |
| **Whole Oat** | 11.5*–37.7 | 8.20–24.0 | 4.00–22.00 | 1.67–7.36 | 1.80–7.00 | 9.70–13.20 | [1,3,10–20] |
| Bran | 24.26 | 11,03 | 21.10* | 11.2 | 9.60–12.0 | 3.00 | |
| Endosperm | 3.60–25.0 | – | – | <1.00 | 1.40–2.30 | 1.80–6.10 | |
| Hull | 65.2 | 19.6–29.8 | 30.8 | 14.8–25.4 | 1.40 | 24.0 | |
| **Whole Barley** | 14.6*–27.1 | 1.90–4.00 | 14,6 | 3.20–3.50 | 2.30–10.50 | 04.20–6.60 | [1–4,6,10,17,18,21,22] |
| Bran | 77.1** | 23.0 | 32.7 | 21.4 | 4.50–7.10 | 7.99–10.26 | |
| Endosperm | 6.48–8.38 | 1.20 | 9.00 | 1.40 | 2.48–2.95 (including water soluble 0.48) | 0.7–2.13 (including water soluble 0.45) | |
| Hull | 59.4 | 19.2–35 | 28.6–33 | 11.5–25.0 | 1.60 | 23.5 | |
| **Whole Rye** | 14.7–20.9 | 2.24–2.80 | 1.90–2.90 | 3.0–4.5 | 1.30–2.20 | 1.28–1.44 | [1,3,4,6,23,24] |

| | | | | | | | |
|---|---|---|---|---|---|---|---|
| Bran | 49.0 | 3.90 | 38.4 | 6.80 | 4.50 | 29.2 | |
| Endosperm | 6.20 | – | 1.50–2.00 | – | 1.54 | 3.65–4.25 (include water soluble 1.64) | |
| **Whole Sorghum** | 7.55–12.3 | 1.40 | 4.00 | 2.40 | 0.1–1.7 | 2.40 | [3,4,25–30] |
| Bran | 8.25–35.10 | 14.9–23.6 | 20.9–37.5 | 0.90–1.40 | 4.50 | 29.2 | |
| Endosperm | 6.00–16.8 | 1.40 | 0.10 | n.d. | – | 5.40 | |
| **Whole Corn** | 10.1 | 2.00 | 7.00 | 1.10 | 0.10 | 4.70 | [4] |
| Bran | 40.5 | 8.90 | 28.6 | 3.00 | 0.20 | 20.7 | |
| Endosperm | 2.60 | – | 2.10 | 0.40 | 0.10 | 1.00 | |
| **Whole Rice** | 12.29 | 3.26 | 11.54 | 4.74 | 0.1–1.6 | 2.86 | [3,7,18,31–34] |
| Bran | 33.9–58.7** | 8.11–15.8 white rice<br>28.6 black rice | 26.4–31.3 | 6.18–11.6 | 0.04–0.21 | 6.82–9.2 | |
| Endosperm | – | – | – | n.d. | – | 1.83 | |

"–" lack of information; n.d.– not detected; *–peeled varieties; **– determined by chemical method; DF – dietary fiber.

## References


1. Rakha, A.; Saulnier, L.; Åman, P.; Andersson, R. Enzymatic fingerprinting of arabinoxylan and β–glucan in triticale, barley and tritordeum grains. *Carbohydr. Polym.* **2012**, *90*, 1226–1234, doi:10.1016/j.carbpol.2012.06.054.
2. Boukid, F. Comprehensive review of barley dietary fibers with emphasis on arabinoxylans. *Bioact. Carbohydr. Diet. Fibre* **2024**, *31*, 100410, doi:10.1016/j.bcdf.2024.100410.
3. Li, W.; Xu, R.; Qin, S.; Song, Q.; Guo, B.; Li, M.; Zhang, Y.; Zhang, B. Cereal dietary Fiber regulates the quality of whole grain products: interaction between composition, modification and processing adaptability. *Int. J. Biol. Macromol.* **2024**, *274*, 133223, doi:10.1016/j.ijbiomac.2024.133223.
4. Knudsen, K.E.B. Fiber and nonstarch polysaccharide content and variation in common crops used in broiler diets. *Poult. Sci.* **2014**, *93*, 2380–2393, doi:10.3382/ps.2014–03902.
5. Kulathunga, J.; Simsek, S. Dietary fiber variation in ancient and modern wheat species: Einkorn, emmer, spelt and hard red spring wheat. *J. Cereal Sci.* **2022**, *104*, 103420, doi:10.1016/j.jcs.2022.103420.
6. Comino, P.; Collins, H.; Lahnstein, J.; Gidley, M.J. Effects of diverse food processing conditions on the structure and solubility of wheat, barley and rye endosperm dietary fibre. *J. Food Eng.* **2016**, *169*, 228–237, doi:10.1016/j.jfoodeng.2015.08.037.
7. Zhong, J.; Xie, H.; Wang, Y.; Xiong, H.; Zhao, Q. Nanofibrillated cellulose derived from rice bran, wheat bran, okara as novel dietary fibers: Structural, physicochemical, and functional properties. *Int. J. Biol. Macromol.* **2024**, *273*, 132902, doi:10.1016/j.ijbiomac.2024.132902.
8. Ma, S.; Wang, Z.; Liu, H.; Li, L.; Zheng, X.; Tian, X.; Sun, B.; Wang, X. Supplementation of wheat flour products with wheat bran dietary fiber: purpose, mechanisms, and challenges. *Trends Food Sci Technol* **2022**, *123*, 281–289, doi:10.1016/j.tifs.2022.03.012.

9. Saroj, R.; Kaur, S.; Malik, M.A.; Puranik, V.; Kaur, D. Thermal processing of wheat bran: effect on the bioactive compounds and dietary fiber. *Bioact. Carbohydr. Diet. Fibre* **2024**, *32*, 100433, doi:10.1016/j.bcdf.2024.100433.
10. Frølich, W.; Åman, P.; Tetens, I. Whole grain foods and health—a scandinavian perspective. *Food Nutr. Res.* **2013**, *57*, 18503, doi:10.3402/fnr.v57i0.18503.
11. Kanwar, P.; Yadav, R.B.; Yadav, B.S. Cross–linking, carboxymethylation and hydroxypropylation treatment to sorghum dietary fiber: Effect on physicochemical, micro structural and thermal properties. *Int. J. Biol. Macromol.* **2023**, *233*, 123638, doi:10.1016/j.ijbiomac.2023.123638.
12. Konakbayeva, D.; Kuspangaliyeva, B.; Rajabzadeh, A.R.; Tabtabaei, S. Separation behavior of sieved endosperm–enriched oat fractions via tribo–electrostatic approach. *Innov. Food Sci. Emerg. Technol .* **2022**, *80*, 103098, doi:10.1016/j.ifset.2022.103098.
13. Nikinmaa, M.; Zehnder–Wyss, O.; Nyström, L.; Sozer, N. Effect of extrusion processing parameters on structure, texture and dietary fibre composition of directly expanded wholegrain oat–based matrices. *LWT* **2023**, *184*, 114972, doi:10.1016/j.lwt.2023.114972.
14. Yang, C.; Li, J.; Luo, T.; Tu, J.; Zhong, T.; Zhang, Y.; Liang, X.; Zhang, L.; Zhang, Z.; Wang, J. Ultrasonic-microwave assisted extraction for oat bran polysaccharides: CHARACTERIZATION and in vivo anti–hyperlipidemia study. *Ind. Crop. Prod.* **2024**, *220*, 119229, doi:10.1016/j.indcrop.2024.119229.
15. Kozan, H.İ.; Sarıçoban, C. Effect of oat bran addition on the survival of selected probiotic strains in turkish fermented sausage during cold storage. *Food Biosci.* **2023**, *54*, 102820, doi:10.1016/j.fbio.2023.102820.
16. Leung, H.; Arrazola, A.; Torrey, S.; Kiarie, E. Utilization of soy hulls, oat hulls, and flax meal fiber in adult broiler breeder hens. *Poult. Sci.* **2018**, *97*, 1368–1372, doi:10.3382/ps/pex434.
17. Neitzel, N.; Eder, M.; Hosseinpourpia, R.; Walther, T.; Adamopoulos, S. Chemical composition, particle geometry, and micro–mechanical strength of barley husks, oat husks, and wheat bran as alternative raw materials for particleboards. *Mater Today Commun.* **2023**, *36*, 106602, doi:10.1016/j.mtcomm.2023.106602.
18. Arzami, A.N.; Ho, T.M.; Mikkonen, K.S. Valorization of cereal by–product hemicelluloses: Fractionation and purity considerations. *Food Res. Int.* **2022**, *151*, 110818, doi:10.1016/j.foodres.2021.110818.
19. Gu, Y.; Qian, X.; Sun, B.; Ma, S.; Tian, X.; Wang, X. Nutritional composition and physicochemical properties of oat flour sieving fractions with different particle size. *LWT* **2022**, *154*, 112757, doi:10.1016/j.lwt.2021.112757.
20. Alfredo Zambrano, J.; Thyagarajan, A.; Sardari, R.R.R.; Olsson, O. Characterization of high arabinoxylan oat lines identified from a mutagenized oat population. *Food Chem.* **2023**, *404*, 134687, doi:10.1016/j.foodchem.2022.134687.
21. Hikawczuk, T.; Szuba–Trznadel, A.; Wróblewska, P.; Wiliczkiewicz, A. Oat hull as a source of lignin–cellulose complex in diets containing wheat or barley and its effect on performance and morphometric measurements of gastrointestinal tract in broiler chickens. *Agriculture* **2023**, *13*, 896, doi:10.3390/agriculture13040896.
22. Park, K.H.; Lee, K.Y.; Lee, H.G. Chemical composition and physicochemical properties of barley dietary fiber by chemical modification. *Int. J. Biol. Macromol.* **2013**, *60*, 360–365, doi:10.1016/j.ijbiomac.2013.06.024.
23. Koj, K.; Pejcz, E. Rye dietary fiber components upon the influence of fermentation inoculated with probiotic microorganisms. *Molecules* **2023**, *28*, 1910, doi:10.3390/molecules28041910.
24. Maina, N.H.; Rieder, A.; De Bondt, Y.; Mäkelä–Salmi, N.; Sahlstrøm, S.; Mattila, O.; Lamothe, L.M.; Nyström, L.; Courtin, C.M.; Katina, K.; et al. Process–induced changes in the quantity and characteristics of grain dietary fiber. *Foods* **2021**, *10*, 2566, doi:10.3390/foods10112566.
25. Adebo, J.A.; Kesa, H. Evaluation of nutritional and functional properties of anatomical parts of two sorghum (*Sorghum bicolor*) varieties. *Heliyon* **2023**, *9*, e17296, doi:10.1016/j.heliyon.2023.e17296.
26. Luna, P.; Risfaheri; Hoerudin; Charalampopoulos, D.; Chatzifragkou, A. fractionation of carbohydrate polymers from indonesian sorghum by–products. *Food Bioprod. Process.* **2022**, *135*, 114–122, doi:10.1016/j.fbp.2022.07.007.
27. de Oliveira, L. de L.; de Oliveira, G.T.; de Alencar, E.R.; Queiroz, V.A.V.; de Alencar Figueiredo, L.F. Physical, chemical, and antioxidant analysis of sorghum grain and flour from five hybrids to determine the drivers of liking of gluten–free sorghum breads. *LWT* **2022**, *153*, 112407, doi:10.1016/j.lwt.2021.112407.
28. Alvarenga, I.C.; Ou, Z.; Thiele, S.; Alavi, S.; Aldrich, C.G. Effects of milling sorghum into fractions on yield, nutrient composition, and their performance in extrusion of dog food. *J. Cereal. Sci.* **2018**, *82*, 121–128, doi:10.1016/j.jcs.2018.05.013.

29. Zhang, Z.; Smith, C.; Li, W. Extraction and modification technology of arabinoxylans from cereal by–products: A critical review. *Food Res. Int.* **2014**, *65*, 423–436, doi:10.1016/j.foodres.2014.05.068.
30. Qiu, S.; Yadav, M.P.; Yin, L. Characterization and functionalities study of hemicellulose and cellulose components isolated from sorghum bran, bagasse and biomass. *Food Chem.* **2017**, *230*, 225–233, doi:10.1016/j.foodchem.2017.03.028.
31. Ma, Q.; Wang, X.; Zhang, R.; Huang, F.; Jia, X.; Dong, L.; Liu, D.; Zhang, M. Structural, physicochemical and functional properties of dietary fiber from black rice bran treated by different processing methods. *Food Biosci.* **2025**, *65*, 106025, doi:10.1016/j.fbio.2025.106025.
32. Ma, Z.–Q.; Zhang, N.; Zhai, X.–T.; Tan, B. Structural, physicochemical and functional properties of dietary fiber from brown rice products treated by different processing techniques. *LWT* **2023**, *182*, 114789, doi:10.1016/j.lwt.2023.114789.
33. Colasanto, A.; Travaglia, F.; Bordiga, M.; Coïsson, J.D.; Arlorio, M.; Locatelli, M. Impact of traditional and innovative cooking techniques on Italian black rice (*Oryza sativa*, L., Artemide cv) composition. *Food Res. Int.* **2024**, *194*, 114906, doi:10.1016/j.foodres.2024.114906.
34. Zhang, D.; Ye, Y.; Wang, L.; Tan, B. Nutrition and Sensory Evaluation of Solid–State Fermented Brown Rice Based on Cluster and Principal Component Analysis. *Foods* **2022**, *11*, 1560, doi:10.3390/foods11111560
